# Quality Assessment of Radiotherapy Health Information on Short-Form Video Platforms of TikTok and Bilibili: Cross-Sectional Study

**DOI:** 10.2196/73455

**Published:** 2025-09-23

**Authors:** Feihang Guo, Guangcheng Ding, Yanzheng Zhang, Xinru Liu

**Affiliations:** 1 The Fifth Affiliated Hospital of Zhengzhou University, 3 Kangfuqian Street, Erqi District, Zhengzhou, China, 86 13623861790

**Keywords:** radiotherapy, RT, short-form videos, information quality, social media, Global Quality Score, DISCERN score, TikTok, Bilibili

## Abstract

**Background:**

Radiotherapy (RT) is a crucial modality in cancer treatment. In recent years, the rise of short-form video platforms has transformed how the public accesses medical information. TikTok and Bilibili, as leading short-video platforms, have emerged as significant channels for disseminating health information. However, there is an urgent need to evaluate the quality and reliability of the information related to RT available on these platforms.

**Objective:**

This study aims to systematically assess the information quality and reliability of RT-related short-form videos on TikTok and Bilibili platforms using the Global Quality Score (GQS) and a modified DISCERN (mDISCERN) evaluation tool, thereby elucidating the current landscape and challenges of digital health communication.

**Methods:**

This study systematically retrieved the top 100 RT-related videos on TikTok and Bilibili as of February 25, 2025. The quality of the videos was assessed using the GQS (1‐5 points) and an mDISCERN scoring system (1‐5 points). Statistical analyses were conducted using the Mann-Whitney *U* test, as well as Spearman and Pearson correlation analyses, to ensure the reliability and validity of the results.

**Results:**

A total of 200 short-form videos related to RT were analyzed, revealing that the overall quality of videos on TikTok and Bilibili is unsatisfactory. Specifically, the median GQS for TikTok was 4 (IQR 3‐4), while for Bilibili, it was 3 (IQR 3‐4). The median mDISCERN scores for both platforms were 3 (IQR 2‐4 and 3‐4, respectively), and no significant differences were observed between the 2 platforms regarding the GQS (*P=*.12) and mDISCERN score (*P=*.10). On TikTok, 53% (53/100) of videos had a GQS of 4 or higher (“good” quality or better). On Bilibili, 45% (45/100) of videos had an mDISCERN score of 4 or higher, indicating “relatively reliable” quality. Videos produced by professionals, institutions, and nonprofessional institutions had significantly higher mDISCERN scores than those made by patients, with statistical significance (*P<*.001, *P<*.001, and *P<*.01, respectively). Furthermore, the correlations between the number of bookmarks and video duration, with mDISCERN scores, were 0.172 (*P=*.02) and 0.192 (*P=*.007), respectively. However, no video variables were found to predict the overall quality and reliability of the videos effectively.

**Conclusions:**

This study revealed that the overall quality of RT-related videos on TikTok and Bilibili is generally low. However, videos uploaded by professionals demonstrate higher information quality and reliability, providing valuable support for patients seeking guidance on health care management and treatment options for cancers. Therefore, improving the quality and reliability of video content, particularly that produced by patients, is crucial for ensuring that the public has access to accurate medical information.

## Introduction

Radiotherapy (RT) plays a crucial role in cancer treatment. It uses high-energy radiation to directly target tumor cells, causing DNA damage that inhibits tumor growth and spread. In recent years, advancements in technology have significantly enhanced the precision and effectiveness of RT, making it a vital treatment option for many patients with cancer [1,2]. Modern RT techniques, such as stereotactic radiosurgery and intensity-modulated radiation therapy, enable precise tumor localization while minimizing damage to surrounding healthy tissue. These techniques use computer-assisted treatment planning to ensure optimal radiation dose distribution [3-5]. Additionally, RT can activate the body’s immune response, enhancing the immune system’s ability to recognize and attack tumor cells. This not only improves treatment outcomes but also reduces side effects and enhances patients’ quality of life [6-9].

As the number of patients with cancer continues to rise each year, cancer treatment regimens, particularly RT, exhibit promising future characteristics [10,11]. As a primary means of obtaining medical information, particularly regarding treatment in the field of cancer, in today's fast-paced society, it is crucial to ensure the quality of videos on short-video platforms. Our research aims to assess the quality of videos providing information related to RT on platforms such as TikTok and Bilibili [12]. As cancer subtypes continue to be explored, treatment modalities have become increasingly diversified [13]. RT, as a core method for cancer treatment, has seen significant advancements globally [1]. With ongoing innovations in the precision and personalization of RT techniques, public awareness of this treatment has also grown [14,15]. China has experienced rapid advancements in the research and application of RT, with a robust body of work emerging in recent years [16]. The integration of technological innovations with clinical practice has continuously enhanced the precision and effectiveness of cancer treatments [17]. The dissemination, promotion, and popularization of RT techniques are crucial for the diagnosis and treatment of various cancers [18].

Despite a marked transition toward electronic health formats, significant disparities in eHealth literacy persist across various populations. These disparities hinder access to health information and services for specific individuals, underscoring the need for targeted interventions to enhance digital competencies and ensure equitable access to eHealth resources [19-21]. People increasingly acquire medical knowledge through web-based channels, including short-video platforms, search engines, and artificial intelligence. To enhance academic rigor, this study focuses on China’s most popular short-video platforms, TikTok and Bilibili. Some global short-video platforms, such as YouTube and Instagram Reels, have a level of video quality and reliability that is as unsatisfactory as that of TikTok and Bilibili [22,23]. As a leading global short-video–hosting platform, TikTok boasts a substantial user base across more than 150 countries, with more than 1.026 billion monthly active users in the Chinese market alone. Meanwhile, Bilibili has also experienced significant user growth due to its unique interactive features and rich content [24-26]. Since the advent of 5G technology, the development of social media platforms has accelerated, establishing them as emerging channels for the dissemination of health information [27]. Research indicates that more than 10 million users engage with health and wellness content on short-video platforms, covering various medical fields, including liver cancer, thyroid cancer, gastric cancer, pancreatic neuroendocrine tumors, and other medical fields [26,28-37]. However, discussions surrounding RT remain scarce in the general population, and the quality of information is often inconsistent. Inaccurate information about RT increases the risk of patients with cancer making misleading decisions based on unreliable sources, making it essential to assess the quality of RT-related videos on social media. To our knowledge, no prior studies have systematically assessed the quality of RT-related content on short-form video platforms, highlighting a significant gap that our research aims to address.

To systematically evaluate the quality and reliability of RT-related short videos, this study recorded and organized the top 100 RT-related videos on TikTok and Bilibili. We used the Global Quality Score (GQS) for quality assessment and the modified DISCERN (mDISCERN) tool for reliability evaluation. Additionally, we analyzed the relationships between video quality and various factors, including video source, content, the number of followers of the uploader, video duration, and interaction metrics (likes, comments, shares, and bookmarks). Our research aimed to evaluate the information quality and reliability of short RT videos on Bilibili and TikTok.

## Methods

### Ethical Considerations

This study involved the analysis of publicly available short-video data from TikTok and Bilibili, which are accessible to the general public. No personal identifying information was collected, and no interaction with individuals occurred; all privacy concerning participants has been preserved. According to the standards proposed by Eysenbach and Till [38], researchers may conduct research in public places or use publicly available information about individuals without obtaining consent. According to the Declaration of Helsinki, this type of research, which uses open-source, publicly available data, does not require ethical approval. Therefore, ethical review was not deemed necessary.

### Eligibility Criteria and Information Sources

Videos included in the study must be relevant to RT treatment. The professionals and institutions possess official influencer verification on short-video platforms, indicating that their credentials have been reviewed by the platforms. The content was sourced from 2 prominent Chinese short-form video platforms: TikTok and Bilibili, which are representative of the majority of short-video platforms in China.

### Search Strategy and Data Collection

In this cross-sectional study, we conducted a comprehensive search for videos related to RT on 2 prominent Chinese short-form video platforms: TikTok [39] and Bilibili [40], using “radiotherapy” as the primary keyword. The search was conducted on February 25, 2025, and the top 100 videos from each platform were selected based on a composite ranking score [26,41]. This score was derived from a combination of video completion rate (proportion of viewers who watched beyond 5 seconds), like rate (proportion of viewers who liked the video), comment rate (proportion of viewers who commented on the video), follow rate (proportion of viewers who followed the uploader), and upload time. Default settings were applied, including no restrictions on upload duration, video length, or search scope, to minimize selection bias.

**Figure 1.**
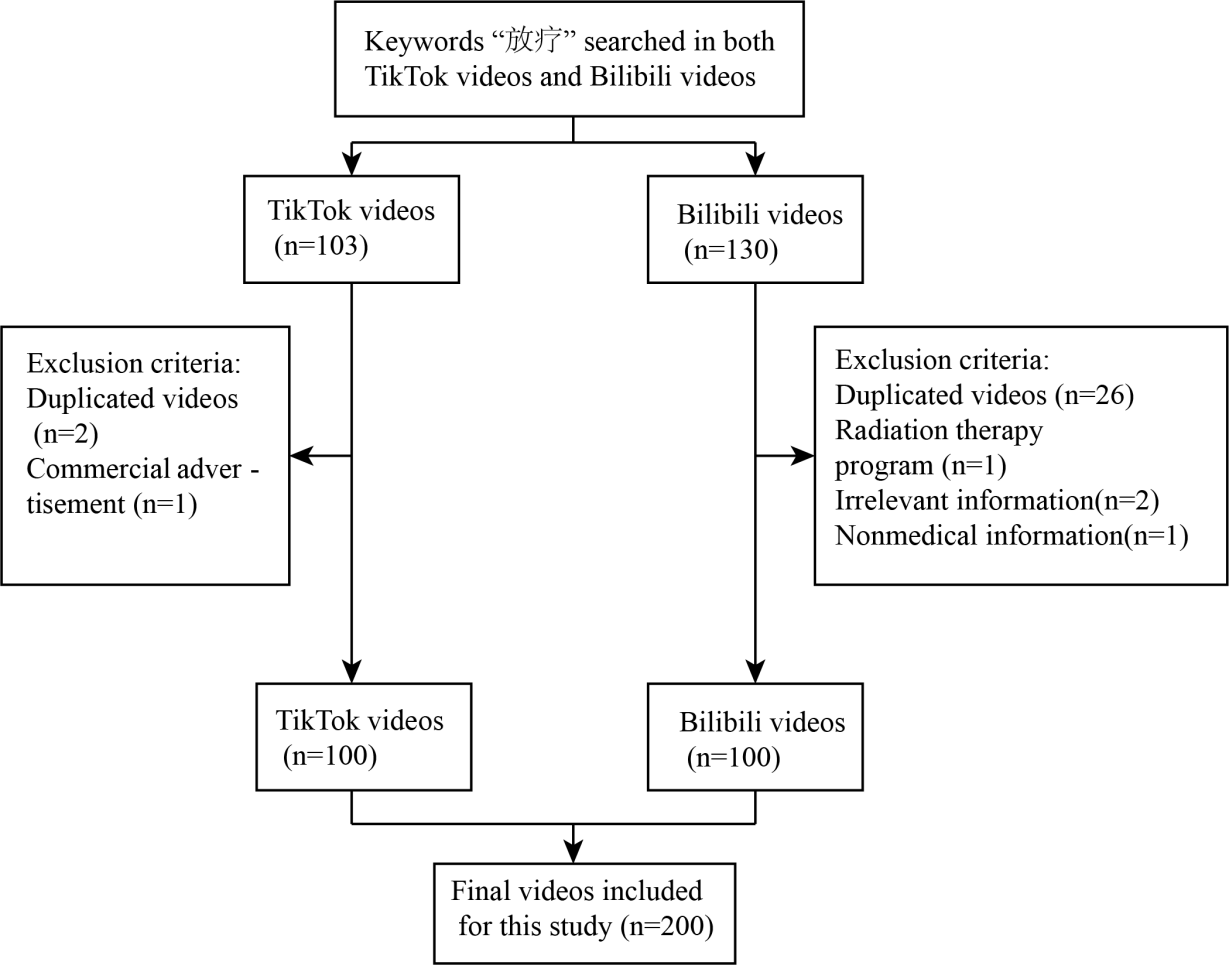
Search strategy and video screening procedure on radiotherapy.

To mitigate the influence of personalized recommendation algorithms, 2 independent viewers uninstalled and reinstalled the applications on their devices, using a clone app to create isolated environments. New accounts were registered and logged in for each platform, ensuring a fresh start for video recommendations. We acknowledge that while this approach may not eliminate personalization, it represents the maximum extent to which patients seeking RT assistance on short-video platforms can be accommodated. Nonmedical videos, duplicates (videos with identical content but different uploaders), videos unrelated to RT, professional RT course videos, and commercial advertisements were systematically excluded until the top 100 videos were identified (Figure 1). Our study limits the number of short videos analyzed on each platform to the top 100, as previous research has demonstrated that videos ranked beyond the top 100 receive very few views, thus having a minimal impact on the study's findings. [26,32,41-45].

The study recorded essential video metadata, including the uploader’s username, identity verification status, follower count, and video metrics, such as likes, comments, saves, shares, and duration. Additionally, video content, source, days since upload, presence of background music, and subtitles were documented.

### Selection Process

Each video was independently reviewed and assessed by the 2 reviewers (FH and YZ). In cases of disagreement, a third viewer was consulted to reach a consensus (XR). All reviewers are radiation oncologists.

### Classification and Assessments of Videos

This study collected and analyzed 100 videos each from Bilibili and TikTok. Each video was meticulously categorized based on its source and content, resulting in four distinct groups for each classification. The source categories included (1) professionals, (2) professional organizations, (3) patients, and (4) nonprofessional institutions. The content categories were (1) personal experiences with RT, (2) educational content on RT, (3) discussions of special cases, and (4) information on RT equipment. Videos from professionals were further classified into (1) radiation oncologists and (2) oncologists. Nonprofessional institutions included (1) science communicators (popular science) and (2) nonprofit or entertainment organizations. Professional organizations referred to official hospital sources or physicians who did not specialize in oncology or RT.

Before evaluating the videos, 2 independent researchers (FH and YZ) reviewed relevant RT guidelines, the GQS, and the mDISCERN tool. The GQS is a widely recognized instrument consisting of 5 criteria, with GQS ranging from 1 to 5, where higher scores indicate better quality of health information videos. The mDISCERN tool was initially developed by Charnock et al [46] to assess written health information and later adapted by Singh et al [47]. Given the medical video context, a modified version of the mDISCERN tool was used to assess the reliability of the content, better aligning it with the specific context of web-based video content. During the review process, researchers evaluated whether each video met the following criteria: clarity, reliable information sources, robustness and fairness, additional references, and statements of uncertainty. Responses to these criteria were recorded as either “yes” (1 point) or “no” (0 points), and a cumulative score (0‐5 points) was calculated, with higher scores indicating greater reliability of the health information presented (Table 1). We conducted a sensitivity analysis by creating a composite index based on GQS and mDISCERN scores with different weights. Under these 2 scoring tools, if the scores of the 2 reviewers are consistent, that score shall be adopted; if not, a third reviewer will review them.

**Table 1.** Description of the Global Quality Score (5-point scale) and the modified DISCERN for evaluating the quality and reliability of videos with Radiotherapy information.

Cumulative Scale		Level	Interpretative descriptor
Global Quality Score			
1		Poor quality	Specifically, the content is illogical, the narrative flow is poor, and most of the information is missing, making it useless for patients.
2		Generally poor quality	The content logic is poor, although some information is listed, more critical information is still missing, and the use of patients is minimal.
3		Moderate quality	Some vital information is adequately discussed.
4		Good quality and flow	Specifically, the video logic is clear and smooth, covering most of the relevant information, which is helpful for patients.
5		Excellent quality and flow	Specifically, the video logic is clear, and the content is very smooth, which is very useful for patients.
Modified DISCERN			
1		Unreliable	Is the video clear, concise, and easy to understand?
2		Less reliable	Are valid sources cited?
3		Fairly reliable	Was the content presented balanced and unbiased?
4		Relatively reliable	Are additional sources of content listed for patient reference?
5		Reliable	Are areas of uncertainty mentioned?

### Statistical Analyses

After conducting a normality test on the data, we determined that it followed a nonparametric distribution. Therefore, descriptive statistics are presented as median (IQR). The Mann-Whitney *U* test was used to assess differences between groups, while Dunn’s Multiple Comparison Test was used for pairwise comparisons. The κ coefficient was calculated to quantify the agreement between the 2 raters. Spearman and Pearson correlation analyses were performed to evaluate the relationships between quantitative variables. A Poisson regression model was used to assess the impact of video variables on predicting video quality and reliability. Statistical significance was defined as a *P* value of <.05. Data analysis and visualization were conducted using R software (version 4.4.1; the R language was developed and is maintained by the R Core Team, and the software is primarily distributed through the Comprehensive R Archive Network) and the Hiplot platform.

## Results

### Video Characteristics

Based on our keyword search, we identified 200 videos for data extraction and analysis, consisting of 100 from TikTok and 100 from Bilibili. The general characteristics of the videos are illustrated in Table 2. This study used the Mann-Whitney *U* test to compare the video characteristics and associated ratings between the Bilibili and TikTok platforms. The results indicated that TikTok significantly outperformed Bilibili in terms of likes median (1129.50 vs 184.00, *Z*=−5.09 *P<.*001), comments (190.00 vs 23.50, *Z*=−5.21; *P<*.001), saves (281.00 vs 60.50, *Z*=−4.27; *P<.*001), and shares (139.00 vs 28.50, *Z*=−4.66; *P<*.001). Additionally, TikTok had a significantly higher follower count (26,500 vs 6,645, *Z*=−3.93; *P*<.001) than Bilibili.

**Table 2.** Characteristics of videos on TikTok and Bilibili.

Variable	Bilibili (n=100), median (IQR)	TikTok (n=100), median (IQR)	Mann-Whitney *U* test	*P* value
Likes	184.00 (43.00-97.50)	1129.50 (300.25-3320.25)	*Z*=−5.09	<.001
Comments	23.50 (2.75-107.00)	190.00 (38.00-528.50)	*Z*=−5.21	<.001
Saves	60.50 (13.50-229.75)	281.00 (62.00-936.50)	*Z*=−4.27	<.001
Shares	28.50 (5.00-92.75)	139.00 (21.75-628.25)	*Z*=−4.66	<.001
Fans	6645.00 (307.50-32,250.00)	26,500.00 (7450.75-117,000.00)	*Z*=−3.93	<.001
Days since upload	604.00 (247.75-1030.00)	70.50 (23.50-126.25)	*Z*=−9.89	<.001
Duration	173.50 (95.75-303.25)	71.50 (39.75-129.25)	*Z*=−6.79	<.001
Global Quality Score	3.00 (3.00-4.00)	4.00 (3.00-4.00)	*Z*=−1.57	.12
M score	3.00 (3.00-4.00)	3.00 (2.00-4.00)	*Z*=−1.66	.10

Although Bilibili demonstrated greater activity in terms of days since video upload (604 vs 70.50, *Z*=−9.89; *P<.*001) and video duration (173.50 vs 71.50, *Z*=−6.79; *P<.*001), no significant differences were observed between the 2 platforms regarding the GQS (*Z*=−1.57; *P=*.12) and mDISCERN score (*Z*=−1.66; *P=*.10).

MultimediaAppendices 1  2, along with Multimedia Appendix 3, illustrate the sources and content types of videos on TikTok and Bilibili. On TikTok, patients with cancer were the most prolific uploaders, contributing 43% (43/100) of the videos, followed by radiation oncologists (24/100, 24%) and medical oncologists (17/100, 17%). Science communicators exhibited the highest levels of user engagement, with a median of 5048 likes (IQR 3,157‐79,500), 393 comments (IQR 160.5‐5005.5), and 1028 saves (IQR 621‐17,500). In terms of content, the majority of TikTok videos focused on sharing personal experiences with RT (51/100, 51%), followed by educational content on RT (33/100, 33%), discussions of special cases (11/100, 11%), and information on RT equipment (5/100, 5%). On Bilibili, patients with cancer also represented the largest group of uploaders, accounting for 27% (27/100) of the videos, followed by other medical professionals or official hospital accounts (22/100, 22%). Science communicators again demonstrated the highest levels of user engagement. Regarding content, educational videos on RT were the most prevalent, comprising 48% (48/100) of the total.

#### Video Quality and Reliability Assessments

**Figure 2.**
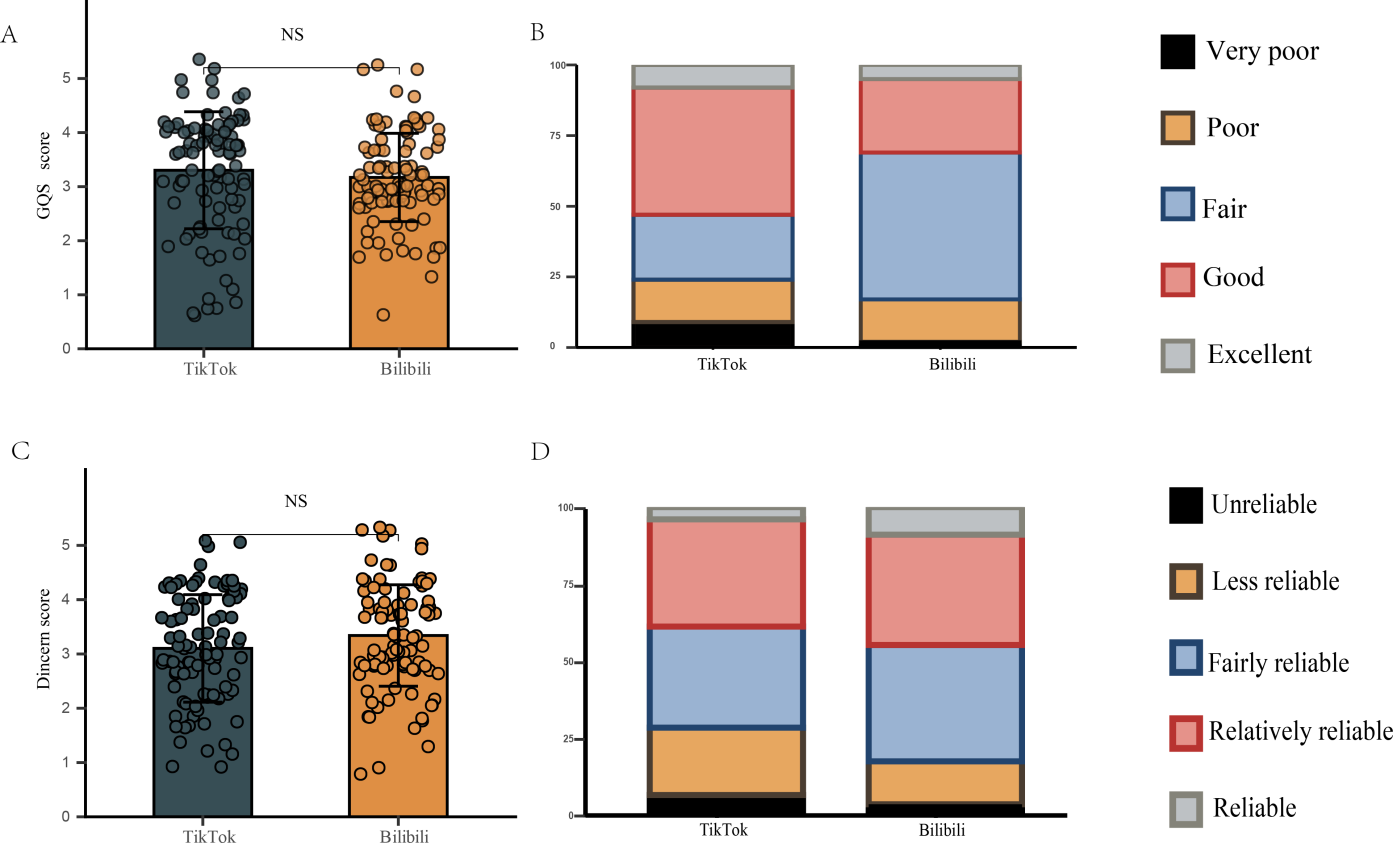
Global Quality Scores, modified DISCERN scores, and quality or reliability distributions of short videos related to radiotherapy on TikTok and Bilibili. (**A**) Comparison of GQS between TikTok and Bilibili videos. (**B**) Proportions of different levels of video quality. (**C**) Comparison of modified DISCERN scores between TikTok and Bilibili videos. (**D**) Proportions of different levels of video reliability. GQS: Global Quality Score; NS: not significant at *P<*.05.

In this study, we evaluated the consistency between the GQS and the mDISCERN score using Cohen κ coefficient for analysis. The results showed that the κ coefficient for GQS was 0.64 and the κ coefficient for the mDISCERN score was 0.68, demonstrating substantial agreement. We conducted a comparative analysis of the GQS and mDISCERN scores for videos on the Bilibili and TikTok platforms. Table 3 and Figure 2A and B revealed that on the Bilibili platform, 31% (31/100) of users rated videos as “good” (4 points) or higher, compared with 53% (53/100) on TikTok. In terms of mDISCERN scores, Table 3 and Figure 2C and Figure 2D revealed that 45% (45/100) of videos on Bilibili received ratings of “relatively reliable” or higher (4 and 5 points), while TikTok had a score of 39% (39/100). Although there were differences (Figure 1), no significant differences were observed between the GQS and mDISCERN scores of TikTok and Bilibili videos (*P=*.12 and *P=*.10, respectively). Multimedia Appendix 4 indicates that the composite index (GQS and mDISCERN weighted differently) for TikTok ranges from 3.109 to 3.261, while for Bilibili, it spans from 3.187 to 3.323.

**Table 3.** Global Quality Score and modified DISCERN scores for TikTok and Bilibili videos related to radiotherapy.

Scale, score	Bilibili (n=100)	TikTok (n=100)	Comparative trend
Global Quality Score			
1 (Poor Quality)	2	9	TikTok
2 (Generally Poor Quality)	15	15	Stable
3 (Moderate Quality)	52	23	Bilibili
4 (Good Quality and Flow)	26	45	TikTok
5 (Excellent Quality and Flow)	5	8	Close
Modified DISCERN			
1 (Unreliable)	3	6	TikTok
2 (Less Reliable)	14	22	TikTok
3 (Fairly Reliable)	38	33	Close
4 (Relatively Reliable)	36	35	Stable
5 (Reliable)	9	4	Bilibili

**Figure 3.**
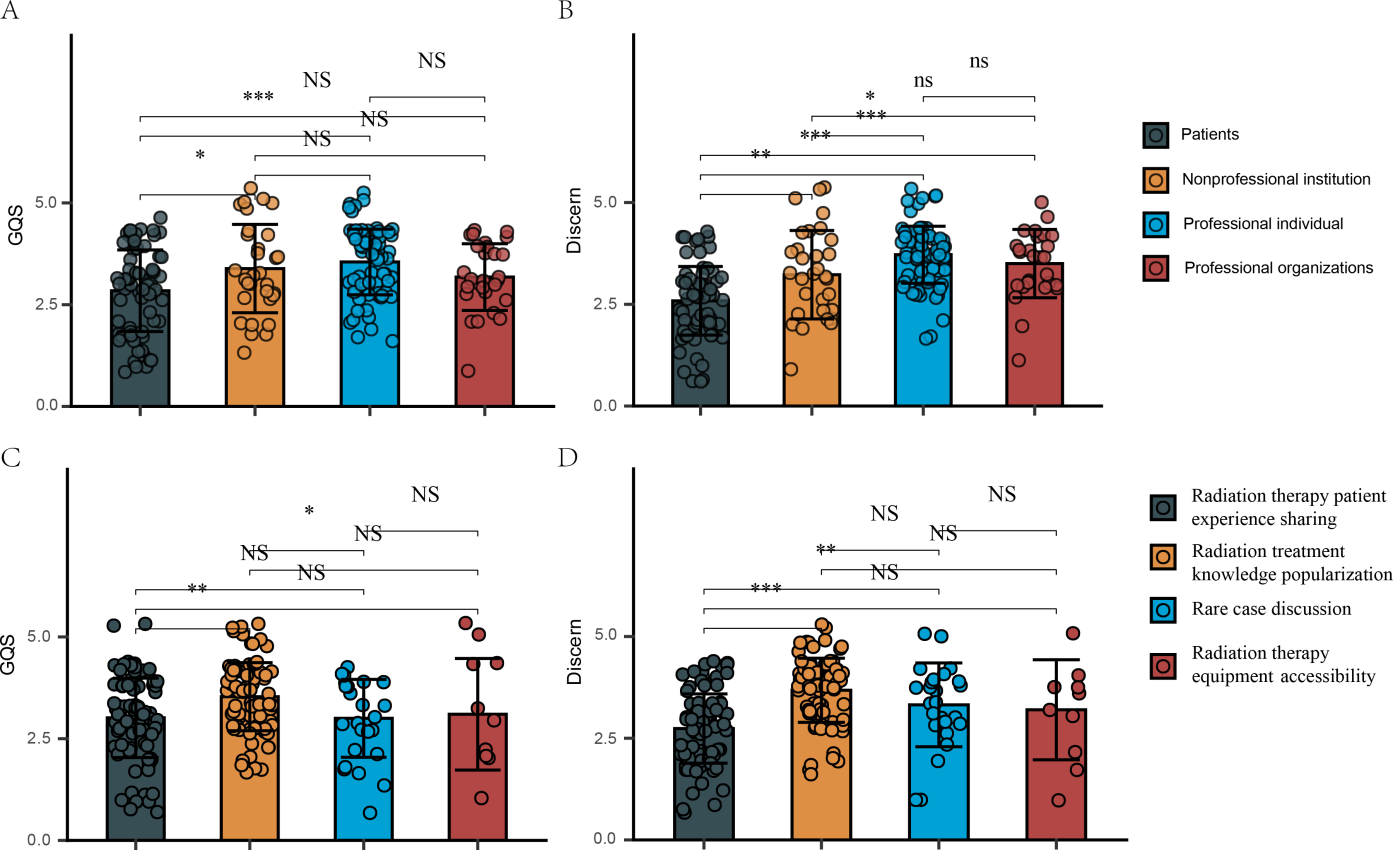
(**A**) GQS from different sources of videos related to radiotherapy (RT). (B) Modified DISCERN scores from different sources of videos related to RT. (C) GQS with different contents of videos related to RT. (**D**) Modified DISCERN scores with different contents of videos related to RT. *P<.05, **P<.01, ***P<.0001. GQS: Global Quality Score; NS: not significant.

We compared the GQS and mDISCERN scores of videos from different sources and with varying content in Figure 3. Figure 3A revealed that the GQS for professionals and nonprofessional institutions was higher than that for patients (*P<.*01 and *P<.*05, respectively). Additionally, Figure 3C revealed that the GQS for videos focused on sharing radiation therapy experiences and special case discussions was lower than that for radiation treatment knowledge popularization (*P*<.01 and *P*<.05, respectively).

Similarly, Figure 3B revealed that the mDISCERN scores for videos from professionals, organizations, and nonprofessional institutions were higher than those for patients (*P<.*001, *P<.*001, and *P<.*01, respectively), with professionals scoring higher than nonprofessional institutions (*P<.*05). Furthermore, Figure 3D revealed that the mDISCERN scores for radiation treatment knowledge popularization and special case discussions were higher than those for radiation therapy experience sharing (*P<.*01and *P<.*01, respectively).

**Figure 4.**
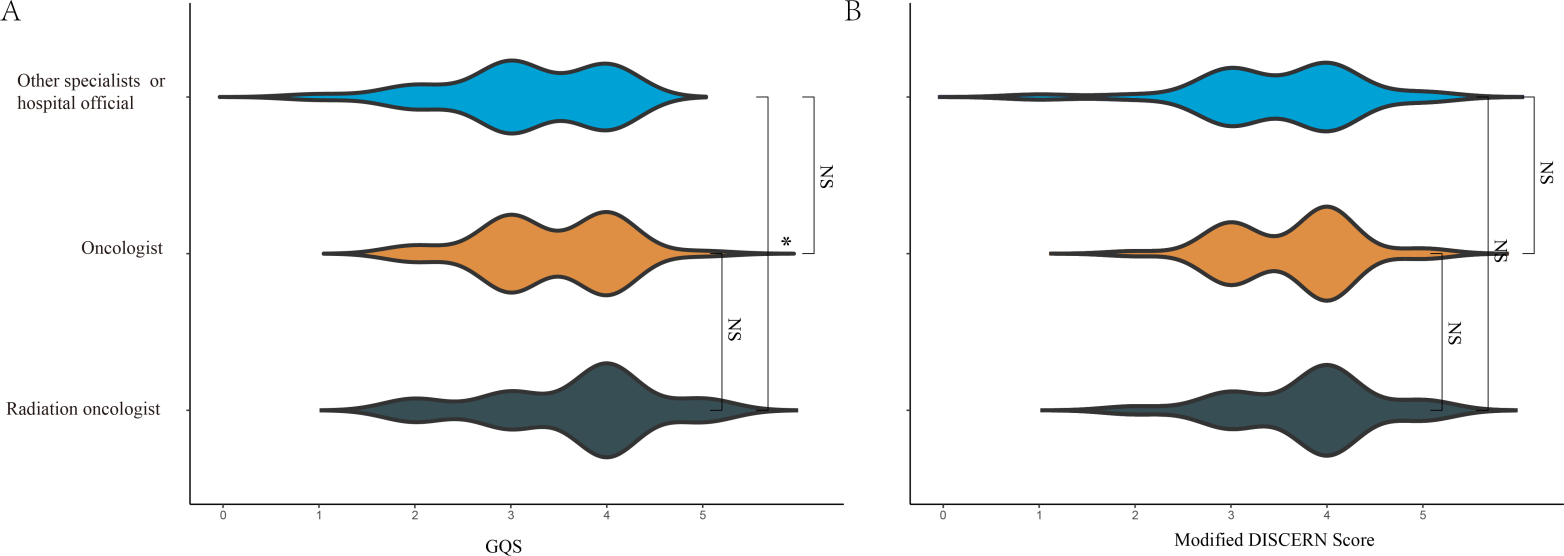
Global Quality Scores (A) and modified DISCERN scores (B) of short videos related to radiotherapy uploaded by different professional individuals. *P<.05. GQS: Global Quality Score; NS: not significant.

To further explore whether different types of professionals and institutions affect the quality and reliability of videos, we further categorized the sources into 3 groups: radiation oncologists, medical oncologists, and other physicians or official hospital sources. Figure 4A revealed that the GQSs for videos from radiation oncologists were higher than those from other physicians or official hospital sources (*P*<.05). However, no significant differences were found among the other source groups (Figure 4B).

**Figure 5.**
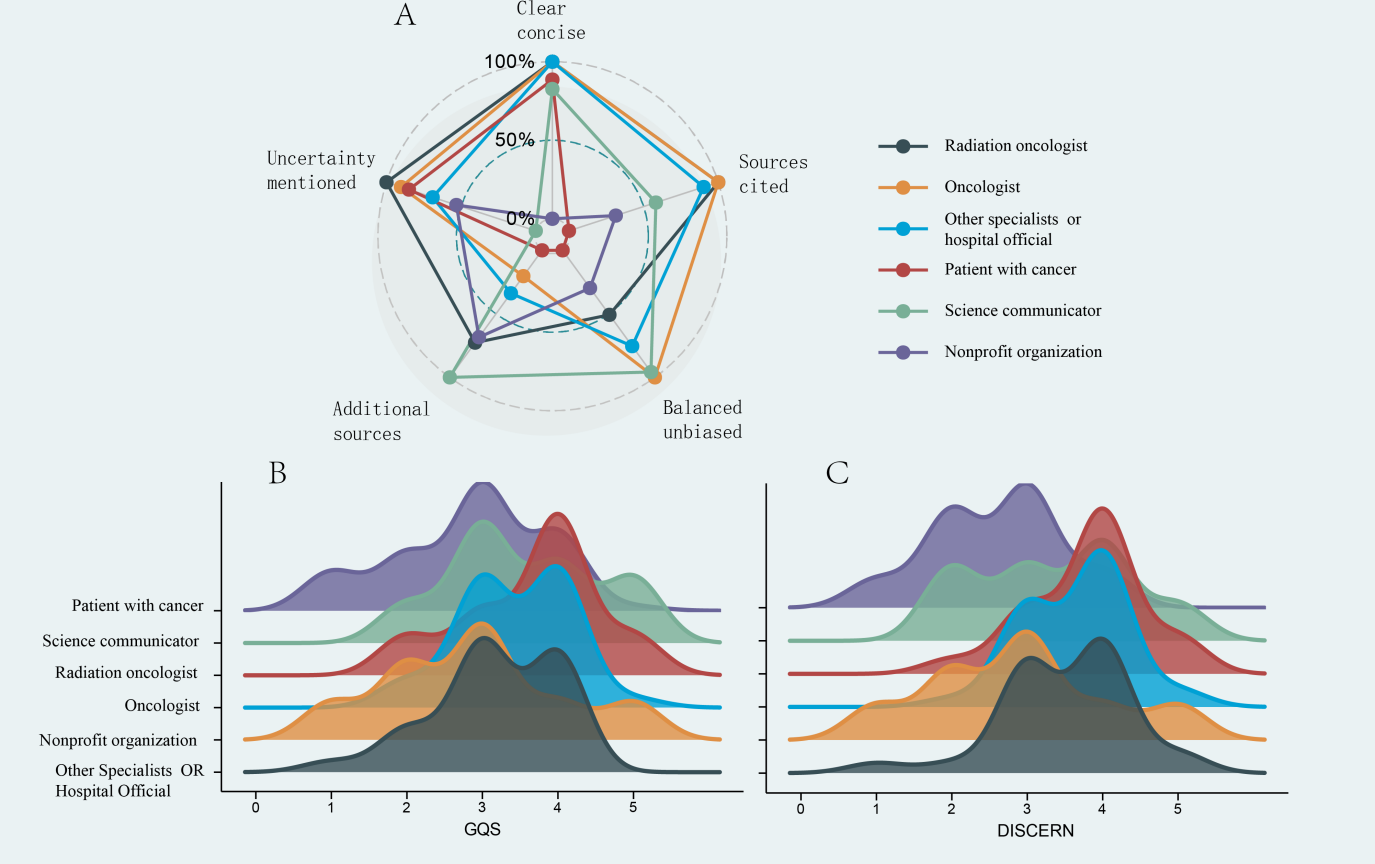
(**A**) Relative strength and balance of mDISCERN scores across different dimensions. (**B**) Overall distribution of Global Quality Scores across different sources. (**C**) Overall distribution of modified DISCERN scores across different sources. GQS: Global Quality Score.

Next, we compared the content comprehensiveness of different video sources using GQS and mDISCERN scores. As shown in Figure 5A, we evaluated the performance of various video sources across multiple dimensions of content comprehensiveness. Key findings include that all video sources consistently scored low on the uncertainty explanation dimension. Figure 5B and Figure 5C revealed that the radiation oncologists generally achieved higher scores than other sources, particularly in terms of video quality and reliability. Patients with cancer and nonprofit organizations exhibited lower scores than other sources. Other physicians or official hospital sources displayed moderate performance.

The GQS and mDISCERN ridge plots indicate that none of the video sources achieved perfect scores, highlighting significant shortcomings in the quality and reliability of radiation therapy videos on short-form platforms. This underscores the need for greater attention from professional doctors and experts to improve the quality and reliability of radiation therapy short videos.

### Correlation Analysis and Poisson Regression Analysis

**Figure 6.**
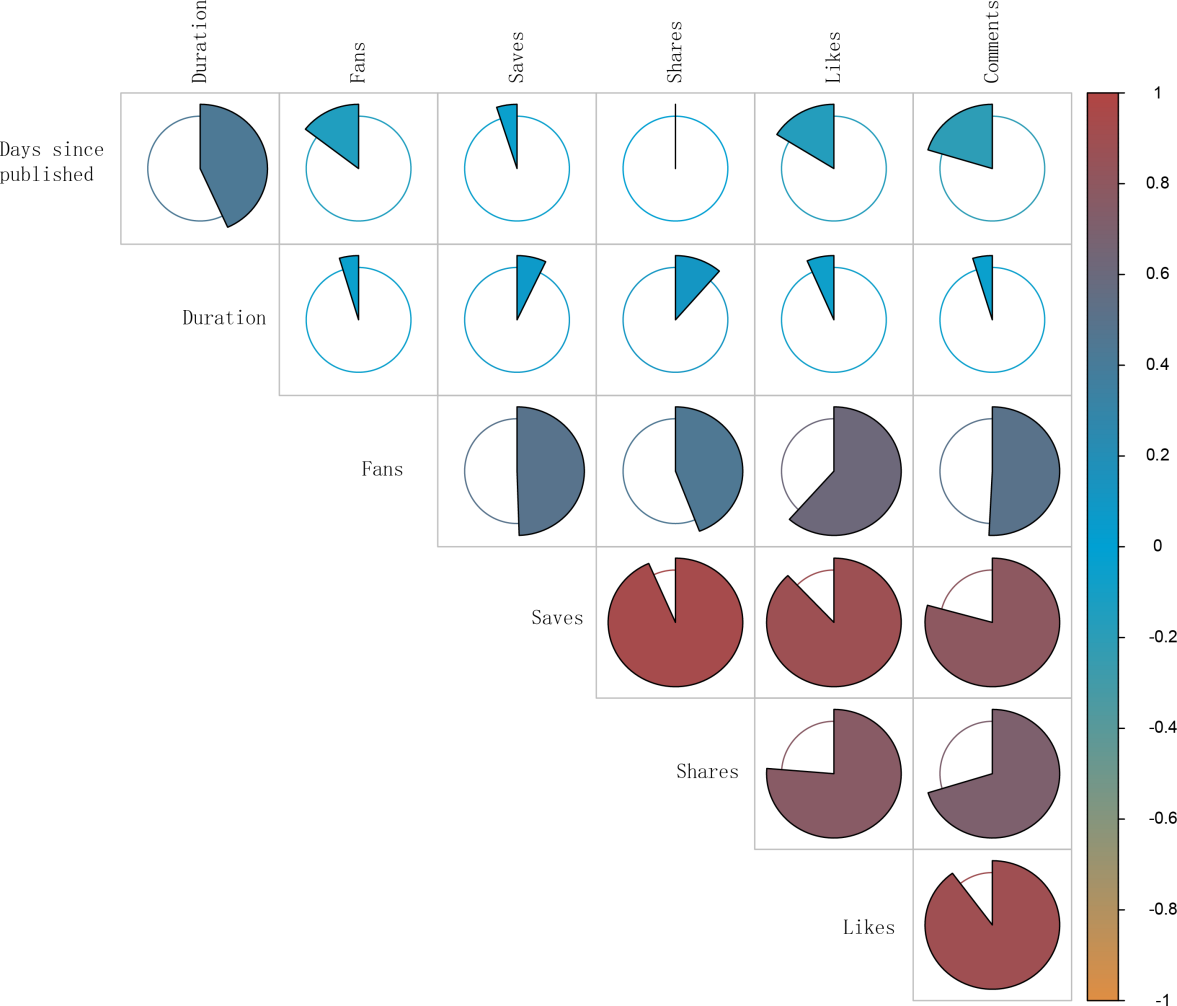
The correlation analysis of relationships between different video variables.

Due to the nonnormal distribution of the data, Spearman correlation analysis was conducted to explore the relationships between different video variables. Table 4 and Figure 6 indicated a robust positive correlation between likes and comments (*r*=0.899; *P<*.01). Additionally, likes showed significant positive correlations with saves (*r*=0.880; *P<*.01) and shares (*r*=0.763; *P<*.01). Comments also had a strong positive correlation with saves (*r*=0.793; *P<*.01) and shares (*r*=0.702; *P<*.01). Notably, days since upload was negatively correlated with likes (*r*=−0.161; *P=*.02) and comments (*r*=−0.203; *P<*.01). There was a positive correlation between duration and days since upload (*r*=0.433; *P<*.01). Furthermore, fans showed significant positive correlations with all interaction metrics: likes (*r*=0.615; *P<*.01), comments (*r*=0.508; *P<*.01), saves (*r*=0.495; *P<*.01), and shares (*r*=0.442; *P<*.01).

**Table 4.** Spearman correlation analysis between the video variables.

Variable		Likes	Comments	Saves	Shares	Days since upload	Duration	Fans
Likes								
*r*		1.000	N/A^a^	N/A	N/A	N/A	N/A	N/A
*P* value		N/A	N/A	N/A	N/A	N/A	N/A	N/A
Comments								
*r*		0.899	1.000	N/A	N/A	N/A	N/A	N/A
*P* value		*<.*01	NA	N/A	N/A	N/A	N/A	N/A
Saves								
*r*		0.880	0.793	1.000	N/A	N/A	N/A	N/A
*P* value		*<.*01	*<.*01	N/A	N/A	N/A	N/A	N/A
Shares								
*r*		0.763	0.702	0.935	1.000	N/A	N/A	N/A
*P* value		*<.*01	*<.*01	*<.*01	N/A	N/A	N/A	N/A
Days since upload								
*r*		−0.161	−0.203	−0.049	−0.008	1.000	N/A	N/A
*P* value		.02	*<.*01	.49	.91	N/A	N/A	N/A
Duration								
*r*		−0.065	−0.047	0.070	0.113	0.433	1.000	N/A
*P* value		.36	.51	.32	.11	*<.*01	N/A	N/A
Fans								
*r*		0.615	0.508	0.495	0.442	−0.145	−0.046	1.000
*P* value		*<.*01	*<.*01	*<.*01	*<.*01	.04	.52	N/A

a Not applicable.

Using Pearson correlation analysis, the relationships between video features and GQS and mDISCERN scores were examined. The results showed that saves and duration had correlations with mDISCERN scores of 0.172 (*P=*.02) and 0.192 (*P=*.007), respectively. However, none of the indicators reached significant levels, as indicated by GQSs (Table 5). Additionally, Table 6 revealed that no significant video variables could predict GQS and mDISCERN scores (all *P*>.05).

**Table 5.** Pearson correlation analysis between video variables and the Global Quality Scores and modified DISCERN scores.

Variable	GQS^a^		Modified DISCERN	
	*R*	*P* value	*R*	*P* value
Likes	0.003	.21	0.137	.054
Comments	0.199	.09	0.055	.44
Saves	0.001	.24	0.172	.02^b^
Shares	0.141	.11	0.045	.53
Days since upload	−0.014	.84	0.074	.30
Duration	0.001	.24	0.192	.007^b^
Fans	0.227	.09	0.101	.16

a GQS: Global Quality Score.

b Significant at *P<*.05.

**Table 6.** Association between video variables and Global Quality Score and mDISCERN score.

Scale, video variable	Relative risk (95% CI)	*P* value
Global Quality Score		
Likes	1.000002 (0.999989‐1.000012)	.79
Comments	0.999995 (0.999940‐1.000047)	.87
Saves	1.000004 (0.999974‐1.000035)	.81
Shares	1.000003 (0.999989‐1.000017)	.67
Days since upload	0.999964 (0.999778‐1.000143)	.70
Duration	1.000139 (0.999959‐1.000297)	.10
Fans	1.000000 (1.000000‐1.000000)	.74
Modified DISCERN		
Likes	1.000000 (0.999986‐1.000011)	.93
Comments	1.000004 (0.999946‐1.000058)	.89
Saves	1.000006 (0.999974‐1.000039)	.73
Shares	1.000000 (0.999984‐1.000013)	.87
Days since upload	1.000032 (0.999850‐1.000207)	.73
Duration	1.000107 (0.999919‐1.000270)	.23
Fans	1.000000 (1.000000‐1.000000)	.74

## Discussion

### Principal Findings

This study systematically reviewed RT-related videos on 2 of the most popular short-form video platforms in China, TikTok and Bilibili, encompassing more than 200 video samples. Overall, the quality of short videos related to RT on both TikTok and Bilibili was found to be unsatisfactory. Although no significant statistical differences in video quality were observed between the 2 platforms (GQS, *Z*=−1.57, *P=*.12; mDISCERN, *Z*=−1.66, *P=*.10), videos produced by professionals or institutions emphasized the dissemination of RT knowledge and techniques, demonstrating higher quality and reliability.

In the field of health care short-video research, various methodologies, inclusion criteria, video variables, sources, and content types are crucial for a comprehensive understanding of the quality and reliability of medical information [48-50]. Some studies share significant similarities with our work, using GQS and mDISCERN tools to assess video quality and reliability, with a focus on platforms such as Douyin and Bilibili, and categorizing video sources into professionals, professional organizations, patients, and nonprofessional institutions [26,32,51,52]. This reflects methodological homogeneity. However, our study introduces methodological innovations by distinguishing between different types of professionals (eg, radiation oncologists and medical oncologists), providing a more nuanced analytical perspective. Notably, most studies have found that videos uploaded by professionals have significantly higher quality than those uploaded by patients, a finding consistent with research on the dissemination of medical information.

Additionally, our study used Poisson regression analysis, indicating that video interaction metrics do not directly predict quality and reliability scores. It must be acknowledged that although some of the Poisson regression models have statistically significant *P* values, the correlations are very weak. For instance, the correlation between the number of saves and duration in the mDISCERN score is only 0.172 and 0.192, which accounts for only a small portion of the variability in the reliability score. Methodologically rigorous, our research ensured data purity through strict retrieval strategies and the removal of duplicate content.

### Quality of the Short Videos on RT

However, the characteristics of short-video formats limit the breadth and depth of content. The brief duration and singular presentation style often prevent viewers from gaining a comprehensive understanding, particularly in educational videos where complex topics such as RT may not be adequately explained within a short time frame. Although we have not directly proven this, we believe that video length may correlate with viewers’ understanding of RT. Previous studies on medical short videos have demonstrated this [53]. Engagement metrics such as likes, comments, and shares are crucial for increasing the visibility of videos on social media platforms. Studies show that higher engagement can lead to improved algorithmic promotion and rankings by platforms, thereby amplifying the reach of the content [54]. However, many qualified physicians do not receive as much engagement as videos uploaded by patients with cancer or entertainment influencers. The prevalence of patients sharing medical videos on RT may lead to a decline in content quality, resulting in partial and incomplete information. This can easily cause misdiagnosis, negative emotions, and skepticism toward professional doctors, ultimately leading to delays in treatment and indecision regarding treatment options, which poses serious risks for patients with cancer [55].

### The Progress of RT Short Videos

Given the strict professional requirements in the field of RT, we advocate for more video uploaders to seek guidance from qualified RT professionals before disseminating information. In our collected videos, medical professionals certified by TikTok and Bilibili are marked with special indicators, and their credentials can be verified through their respective hospitals’ official websites. This enhances the reliability of the videos and fosters greater trust in RT’s knowledge among viewers. RT professionals must not only possess a solid medical foundation but also use clear visual metaphors to break down complex processes into coherent and understandable steps. This approach can reduce the monotony of RT short videos and use relatable analogies that connect professional terminology with everyday understanding. Additionally, integrating interactive elements and evidence-based visual aids can significantly enhance viewers’ comprehension and retention of medical knowledge, effectively disseminating information within a short time frame.

#### Practical Significance

This study provides essential empirical evidence for assessing the quality and reliability of RT-related medical knowledge dissemination on short-form video platforms. By systematically analyzing the quality and reliability of RT videos on TikTok and Bilibili, we have highlighted significant disparities in how the public accesses medical information [37,56,57].

Our findings fill a critical gap in the existing literature and offer valuable insights for health care professionals and regulators of short-video content. Notably, China has introduced the world’s first health promotion guidelines for scientific communication on short-video platforms, marking a significant advancement in this field [37,58,59].

We recommend that platforms implement stricter content verification policies, including mandatory certifications for health-related content creators and a vetting process by licensed professionals. Additionally, prioritizing content from verified medical experts is essential. We also advocate for digital literacy programs that empower patients to critically assess web-based health information, alongside clear guidelines for content creators to ensure adherence to best practices in medical communication. Engaging stakeholders, including platform developers, health care institutions, and public health agencies, will be crucial in collaboratively implementing these measures and improving public health literacy, ultimately contributing to the advancement of global RT practices.

Future research should explore the effectiveness of different types of RT content, develop more precise assessment tools for evaluating the quality of RT content, and investigate the long-term impacts of short-video platforms on public acquisition of medical knowledge and health behaviors. This study represents a crucial step toward improving the quality of medical information in the digital age, providing a comprehensive framework for addressing challenges in web-based health communication.

### Strengths and Limitations

The study demonstrates unique strengths in several aspects. First, the research design systematically evaluated RT-related short videos on TikTok and Bilibili platforms. This approach not only ensures data comprehensiveness but also enhances the representativeness of the research findings. Second, the study conducted a comprehensive assessment of video quality and reliability using the GQS and the mDISCERN tool [26,28,31]. This dual assessment approach increases the credibility of the results, allowing for a more accurate evaluation of video content quality.

In terms of data analysis strategies, this study used nonparametric statistical methods to accommodate the distribution characteristics of the data, ensuring the validity of the analytical results [30]. Additionally, Spearman and Pearson correlation analyses further elucidated the relationships between video features and quality scores, providing deeper insights into the factors influencing short-video content [26]. The application of Poisson regression models offered predictive capabilities regarding the potential relationships between video variables and quality scores, thereby enhancing the depth of the research. Notably, this is the first study in China to analyze the quality of RT-related short videos across 2 social media platforms [60].

However, our study has several limitations that affect the generalizability and external validity of the results. First, the inclusion criteria excluded videos from professional RT courses, meaning that the findings cannot be applied to the quality and reliability assessment of such educational content.

Second, the cross-sectional design of the study restricts the generalizability of the results. Cross-sectional studies provide data at a specific point in time, but they lack long-term observations of trends. Therefore, future research should use longitudinal designs to validate our findings and ensure the stability and reliability of the results. Additionally, the primary focus of this study was on Chinese users, using the Chinese versions of TikTok and Bilibili. Caution should be exercised when applying our findings to other countries and populations, as cultural and social background differences may influence the acceptance and dissemination of video content. Although we selected the top 100 videos from each platform, which represents a small proportion, we believe that this sample is sufficiently representative, as videos beyond the top 100 did not significantly impact the analysis [61-63]. Furthermore, specific subgroup categories (eg, nonprofit organizations and RT equipment promotion) had small sample sizes, which may introduce inaccuracies.

Despite the potential biases that may exist in this study, such as reviewer bias, selection bias, sampling bias, measurement bias, and content bias, we formulated systematic screening criteria. We conducted cross-platform data collection to ensure the diversity and comprehensiveness of the included videos. Furthermore, all reviewers strictly followed the GQS and mDISCERN guidelines for independent scoring, which a third reviewer (XR) subsequently audited, achieving good κ consistency. The final selection of videos not only covers most of the high-quality content on the topic but also possesses good representativeness. Therefore, we believe that the impact of these potential biases on the research outcomes is negligible, providing a solid foundation for the conclusions of this study. Finally, as an observational study, this research can detect associations only between variables and cannot establish causal relationships.

### Conclusions

This study systematically evaluated the quality and reliability of RT-related short videos on 2 major short-video platforms in China, TikTok and Bilibili. By analyzing 200 video samples, we found that the GQS of most videos was unsatisfactory. On the Bilibili platform, 69% (69/100) of the videos received a GQS of 3 or below, while on TikTok, this figure was 47% (47/100). In terms of mDISCERN, the 2 platforms had scores of 55% (55/100) and 61% (61/100), respectively, for scores of 3 or below. Although TikTok outperformed Bilibili in user engagement, there were no significant differences in video quality scores (GQS) and mDISCERN scores between the 2 platforms.

Therefore, we conclude that while short-video platforms have the potential to disseminate medical knowledge, the quality of the content urgently needs improvement. We recommend that relevant platforms and institutions enhance oversight of short-video content to ensure the accuracy and scientific integrity of the information presented. Additionally, encouraging RT professionals to actively participate in creating short videos will help improve public understanding and trust in RT knowledge.

## Supplementary material

10.2196/73455 Multimedia Appendix 1 Characteristics of the videos across sources and content in TikTok.

10.2196/73455 Multimedia Appendix 2 Characteristics of the videos across sources and content in Bilibili.

10.2196/73455 Multimedia Appendix 3 Percentage of videos on radiotherapy from different sources and with varying contents in TikTok and Bilibili. (A) Sources of TikTok videos. (B) Sources of Bilibili videos. (C) Content types of TikTok videos. (D) Content types of Bilibili videos.

10.2196/73455 Multimedia Appendix 4 The composite index (Global Quality Score and modified DISCERN are weighted differently).

## Abbreviations

GQS: Global Quality Score
RT: radiotherapy
