# Supplementary material for: Quality Assessment of Radiotherapy Health Information on Short-Form Video Platforms of TikTok and Bilibili: Cross-Sectional Study
Source: JMIR Cancer. 2025 Sep 23;11:e73455. doi: 10.2196/73455 (PMC12456845; doi:10.2196/73455)
Supplement: Multimedia Appendix 1 [file cancer-v11-e73455-s001.doc]

**Multimedia Appendix 1. Characteristics of the Videos Across Sources and Content in TikTok.**

| Variable | Likes | Comments | Saves | Shares | Days since upload | Duration | Fans |
| --- | --- | --- | --- | --- | --- | --- | --- |
| Video sources(n=100),median (IQR) | | | | | | | |
| Radiation Oncologist (n = 24) | 644.50 (158.00, 1425.75) | 45.50 (12.50, 178.00) | 206.00 (35.25, 637.25) | 131.00 (24.75, 420.00) | 80.50 (57.75, 122.75) | 84.00 (46.75, 116.25) | 24000.00 (5459.50, 70750.00) |
| Oncologist(n = 17) | 399.00 (284.00, 1755.00) | 38.00 (9.00, 115.00) | 123.00 (65.00, 569.00) | 127.00 (10.00, 566.00) | 84.00 (24.00, 105.00) | 64.00 (52.00, 97.00) | 45000.00 (15000.00, 103000.00) |
| Other Specialists OR Hospital Official(n = 6) | 2906.00 (1580.75, 4436.75) | 249.50 (207.00, 540.25) | 851.00 (605.50, 1257.00) | 512.50 (212.25, 1309.25) | 242.50 (59.75, 264.75) | 110.00 (48.00, 165.25) | 134500.00 (43000.00, 189250.00) |
| Cancer Patient(n = 43) | 1249.00 (320.50, 3555.00) | 286.00 (147.50, 601.00) | 180.00 (63.00, 672.50) | 87.00 (18.00, 579.00) | 66.00 (24.50, 138.00) | 59.00 (28.50, 173.50) | 11000.00 (3436.50, 46000.00) |
| Science Communicator(n = 7) | 5048.00 (3157.00, 79500.00) | 393.00 (160.50, 5005.50) | 1028.00 (621.00, 17500.00) | 670.00 (540.00, 20500.00) | 64.00 (33.00, 76.50) | 107.00 (76.00, 150.00) | 622000.00 (409500.00, 1097000.00) |
| Nonprofit Organization(n = 3) | 3520.00 (3350.50, 17260.00) | 707.00 (382.00, 3305.00) | 1664.00 (1017.50, 2365.50) | 752.00 (452.00, 49876.00) | 5.00 (3.00, 20.50) | 18.00 (12.50, 30.00) | 1536000.00 (768000.50, 4020000.00) |
| Video content(n=100),median (IQR) | | | | | | | |
| Radiation Therapy Patient Experience Sharing(n = 51) | 1288.00 (334.50, 3569.50) | 276.00 (97.00, 616.00) | 261.00 (89.50, 906.50) | 151.00 (21.50, 690.50) | 67.00 (28.50, 138.00) | 71.00 (41.00, 159.00) | 13000.00 (3436.50,48000.00) |
| Radiation Treatment Knowledge Popularization(n = 33) | 791.00 (161.00, 2057.00) | 40.00 (11.00, 144.00) | 323.00 (37.00, 936.00) | 160.00 (31.00, 542.00) | 84.00 (57.00, 122.00) | 90.00 (56.00, 125.00) | 60000.00 (7517.00, 158000.00) |
| Rare Case Discussion(n = 11) | 1174.00 (234.00, 3653.00) | 147.00 (12.00, 392.00) | 170.00 (44.00, 526.50) | 97.00 (10.00, 174.00) | 22.00 (11.00, 101.50) | 37.00 (21.50, 43.00) | 45000.00 (31500.00, 103000.00) |
| Radiation Therapy Equipment Accessibility(n = 5) | 4176.00 (3405.00, 52000.00) | 1055.00 (233.00, 3681.00) | 3397.00 (708.00, 15000.00) | 2720.00 (670.00, 14000.00) | 33.00 (4.00, 79.00) | 76.00 (10.00, 147.00) | 412000.00 (70000.00, 1097000.00) |
