# Supplementary material for: Quality Assessment of Radiotherapy Health Information on Short-Form Video Platforms of TikTok and Bilibili: Cross-Sectional Study
Source: JMIR Cancer. 2025 Sep 23;11:e73455. doi: 10.2196/73455 (PMC12456845; doi:10.2196/73455)
Supplement: Multimedia Appendix 2 [file cancer-v11-e73455-s002.doc]

**Multimedia Appendix 2. Characteristics of the Videos Across Sources and Content in Bilibili.**

| Variable | Likes | Comments | Saves | Shares | Days since upload | Duration | Fans |
| --- | --- | --- | --- | --- | --- | --- | --- |
| Video sources(n=100),median (IQR) | | | | | | | |
| Radiation Oncologist (n = 24) | 50.00 (4.25, 95.75) | 1.50 (0.00, 16.50) | 55.00 (3.50, 118.75) | 24.00 (1.25, 59.25) | 247.50 (185.75, 1184.50) | 160.00 (91.25, 492.75) | 149.00 (114.50, 2309.50) |
| Oncologist(n = 17) | 34.00 (12.75, 167.00) | 3.00 (0.75, 49.50) | 13.00 (3.00, 69.50) | 11.50 (2.75, 55.50) | 583.50 (376.25, 892.00) | 108.50 (88.50, 148.00) | 12,113.50 (179.50, 68,000.00) |
| Other Specialists OR Hospital Official(n = 6) | 428.00 (118.50, 771.25) | 64.00 (8.25, 160.50) | 88.50 (19.00, 278.00) | 46.00 (7.50, 146.25) | 695.00 (253.75, 1,163.50) | 205.00 (101.50, 321.50) | 74,500.00 (28,000.00, 156,750.00) |
| Cancer Patient(n = 43) | 262.00 (145.00, 701.00) | 37.00 (22.00, 72.50) | 48.00 (18.00, 112.50) | 12.00 (5.00, 54.00) | 602.00 (485.50, 822.50) | 204.00 (143.50, 288.00) | 2,132.00 (1,529.00, 13,000.00) |
| Science Communicator(n = 7) | 673.00 (50.75, 4,995.25) | 66.50 (2.75, 404.25) | 241.50 (100.50, 568.00) | 79.50 (37.00, 435.00) | 622.00 (132.50, 1,123.00) | 145.50 (81.75, 230.25) | 2,225.00 (202.00, 20,500.00) |
| Nonprofit Organization(n = 3) | 70.00 (7.00, 2,811.00) | 3.00 (0.00, 858.00) | 110.00 (10.00, 4,641.00) | 85.00 (9.00, 422.00) | 1,092.00 (248.00, 1,236.00) | 844.00 (218.00, 1,094.00) | 14,000.00 (104.00, 465,000.00) |
| Video content(n=100),median (IQR) | | | | | | | |
| Radiation Therapy Patient Experience Sharing(n = 51) | 210.00 (59.00, 589.00) | 25.00 (12.00, 66.00) | 46.00 (15.00, 113.00) | 11.00 (5.00, 60.00) | 606.00 (484.00, 868.00) | 184.00 (114.00, 276.00) | 1,898.00 (702.00, 7,847.00) |
| Radiation Treatment Knowledge Popularization(n = 33) | 108.00 (15.75, 787.50) | 8.50 (1.75, 122.00) | 85.50 (11.75, 260.50) | 46.00 (8.25, 144.75) | 677.00 (280.50, 1,209.75) | 159.50 (81.75, 363.00) | 8,693.50 (149.00, 57,250.00) |
| Rare Case Discussion(n = 11) | 474.00 (167.00, 772.25) | 81.50 (24.25, 136.50) | 35.00 (19.00, 198.75) | 17.00 (7.00, 133.00) | 369.00 (74.25, 638.00) | 203.50 (108.75, 325.25) | 68,000.00 (24,500.00, 126,000.00) |
| Radiation Therapy Equipment Accessibility(n = 5) | 146.00 (6.00, 16,000.00) | 26.00 (0.00, 837.00) | 122.00 (5.00, 4,130.00) | 55.00 (2.00, 422.00) | 64.00 (59.00, 248.00) | 158.00 (137.00, 193.00) | 19,000.00 (14,000.00, 25,000.00) |
