# Supplementary figures and images for: Quality Assessment of Radiotherapy Health Information on Short-Form Video Platforms of TikTok and Bilibili: Cross-Sectional Study
Source: JMIR Cancer. 2025 Sep 23;11:e73455. doi: 10.2196/73455 (PMC12456845; doi:10.2196/73455)

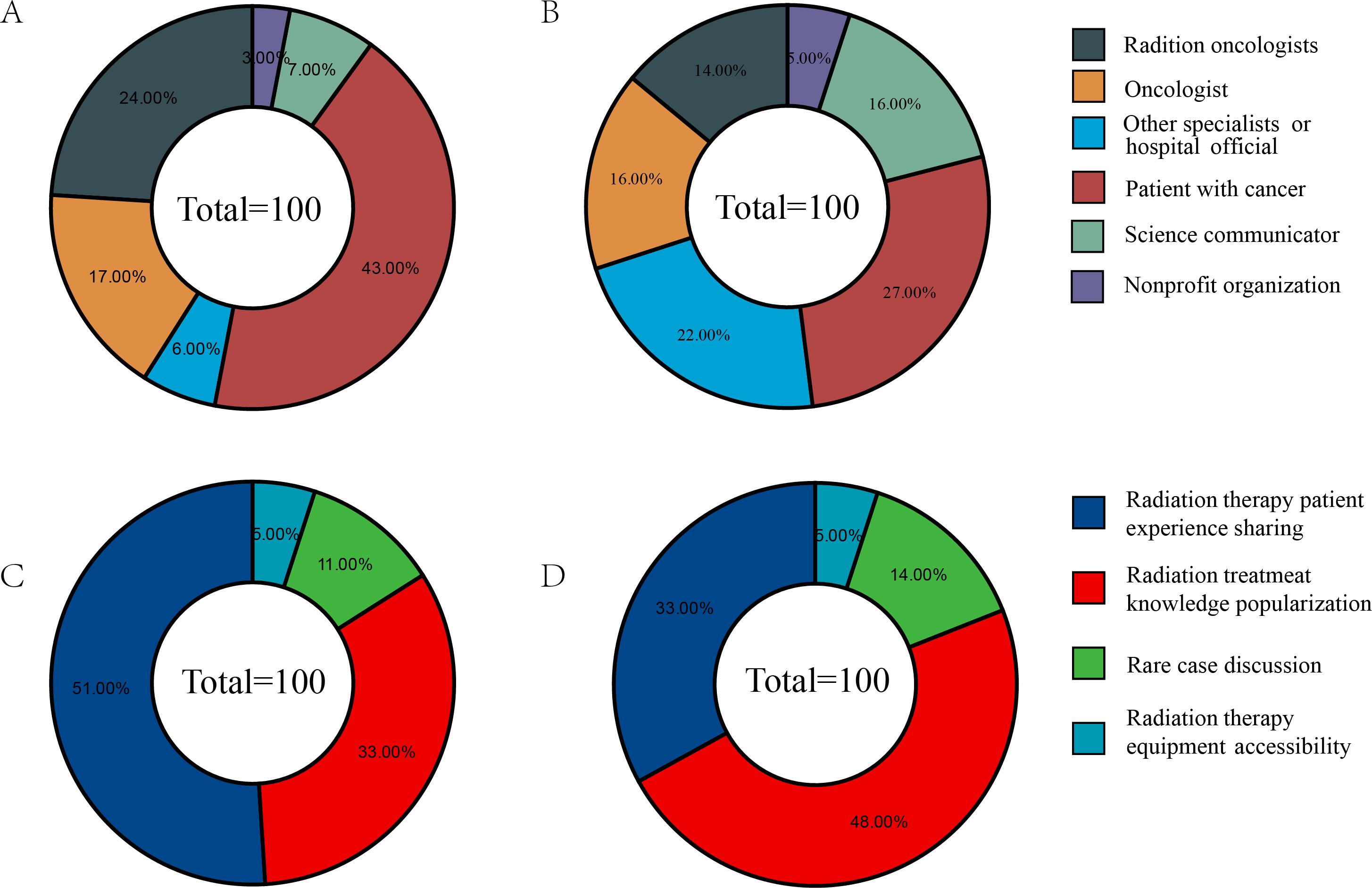

Supplement: Multimedia Appendix 3 [file cancer-v11-e73455-s003.jpg]
